# Semen Quality Measures in Hookah and Cigarette Smokers Compared to Nonsmokers

**DOI:** 10.1155/tswj/3380445

**Published:** 2025-02-17

**Authors:** Jehan Hamadneh, Aseel A. Al-Zenati, Saleem A. Banihani

**Affiliations:** ^1^Department of Obstetrics and Gynecology, Faculty of Medicine, Jordan University of Science and Technology, Irbid, Jordan; ^2^Department of Medical Laboratory Sciences, Faculty of Applied Medical Sciences, Jordan University of Science and Technology, Irbid, Jordan

**Keywords:** cigarette, semen, smoking, sperm count, sperm motility

## Abstract

**Background:** The relationship between smoking and human health is a well-researched and continuously evolving field. The impact of smoking on semen quality, and consequently on male fertility, has also been explored, though most studies have primarily focused on cigarette smoking rather than hookah smoking.

**Objective:** In this study, we aimed to investigate and compare the effects of hookah and cigarette smoking on semen parameters in a sample of Jordanian males.

**Methods:** A total of 558 participants were prospectively recruited, including 300 cigarette smokers, 95 hookah smokers, and 163 nonsmokers (control). Semen analysis was performed approximately 1 h after ejaculation following the World Health Organization guidelines (2021).

**Results:** Interestingly, semen volume was significantly decreased in hookah smokers (*p* = 0.0097) but not in cigarette smokers when compared to the control group. No significant differences were observed in semen volume, progressive sperm motility, total motility, sperm count, sperm morphology (*p* = 0.2714, *p* = 0.8752, *p* = 0.6671, *p* = 8614, and *p* = 0.9261, respectively), and sperm vitality between hookah and cigarette smokers. Furthermore, except for semen volume, these semen parameters were not statistically different in both tested groups when compared to the control group.

**Conclusions:** Hookah smokers demonstrated lower semen volume compared to the control group. Additionally, no significant differences were found in sperm count, percentage of sperm motility, normal forms of sperm, and sperm vitality between hookah and cigarette smokers or between these groups and the control group.

## 1. Introduction

Overall, there is an established correlation between smoking, particularly cigarette smoking, and infertility in both genders [1–3]. Worldwide, from 1990 to 2015, the age-standardized prevalence of daily smoking was approximately 25% for men and 5.4% for women [4]. In 2019, a study conducted in partnership with the World Health Organization (WHO) revealed that Jordan had one of the highest smoking rates globally, with tobacco use reported in approximately 66% of men and 17% of women [5]. In the PubMed database, there are more than 150 published articles that link, however, sometimes indirectly, between cigarette smoking and male infertility. And it is worth mentioning that this number has dramatically increased in the last 10 years. On the other hand, smoking was found to negatively impact the assisted reproductive technologies for couples who are trying to conceive [6]. For example, it has been shown that smokers require approximately twice the number of in vitro fertilization (IVF) attempts to conceive as nonsmokers [6].

However, in 2018, the Practice Committee of the American Society for Reproductive Medicine summarized that even though there is fair evidence that semen fertility characteristics and sperm function are lower in cigarette smokers compared to nonsmokers, still, smoking has not yet been decisively found to decrease fertility of males [6]. Therefore, there is still a need to conduct further research studies to fill this important gap in knowledge. Here, as yet, it is worth stating that the estimated public knowledge of the smoking risk of infertility is approximately 22% [6].

Hookah is a waterpipe that is used to smoke, especially mixtures of tobacco that come in varied flavors. In general, it works by passing charcoal-heated air across the flavored mixture of tobacco via pierced aluminum foil and finally through a bowl filled with water [7]. The smoker then inhales the smoke through a rubber hose connected to a mouthpiece. Products of hookah tobacco come in different pleasant flavors, such as mint, apple, cherry, watermelon, coconut, chocolate, cappuccino, and licorice [7]. It is worth mentioning that there are three used types of hookah tobacco, Tumbak, Mouassal, and Jurak, and each type contains different chemical ingredients [8].

In general, the majority of the published work that links smoking to male infertility discusses this link, particularly in the context of cigarette smoking, but not hookah smoking. For example, retrospectively, cigarette smoking was found to have a significant effect on sperm concentration [9]. A previous study in China (2000) showed that long-term and heavy smoking negatively affects sperm parameters (concentration, motility, and viability) [10]. Also, cigarette smoking was found to significantly alter the DNA methylation patterns of sperm [11, 12]. In addition, a positive correlation was observed between cigarette smoking and sperm DNA fragmentation, which consequently affects sperm function [13, 14]. Alternatively, a study in 2014 by de Jong et al. revealed no significant effect of cigarette smoking on semen parameters [15].

On the other hand, up to the present time, the effect of hookah smoking on semen quality and male infertility is very limited and is poorly examined. A study in 2020 conducted by Albeitawi et al. revealed a decline in the mean values of some semen parameters (semen volume, normal sperm morphology, and progressive motility of sperm) in hookah smokers compared to nonsmokers; however, this decline was not statistically significant [16]. In addition, recent studies suggest that while hookah smoking may not significantly impact conventional sperm parameters, it could have detrimental effects on DNA integrity, oxidative status, and nuclear protein levels of spermatozoa. This highlights the need for further investigation into the differences in semen quality between hookah smokers, cigarette smokers, and nonsmokers [17].

Therefore, in this work, we hypothesized that there may be a variation in semen quality between hookah smokers and cigarette smokers along with a variation between these groups when comparing them with nonsmokers. Accordingly, in this work, we aimed to compare the main semen quality parameters between hookah smokers and cigarette smokers in Jordanian males and refer each group to a matched control (nonsmokers). To do this, we have designed a prospective comparative study to obtain more reliable data.

## 2. Materials and Methods

### 2.1. Study Design and Participants

This study is a prospective observational study conducted between January 2018 and April 2023 in the IVF center at King Abdullah University of the Jordan University of Science and Technology, Jordan. Recruited participants were the men who attended the andrology laboratory and had seminal fluid analysis (*n* = 558) for assisted reproductive technologies such as IVF or intrauterine insemination.

**Figure 1:**
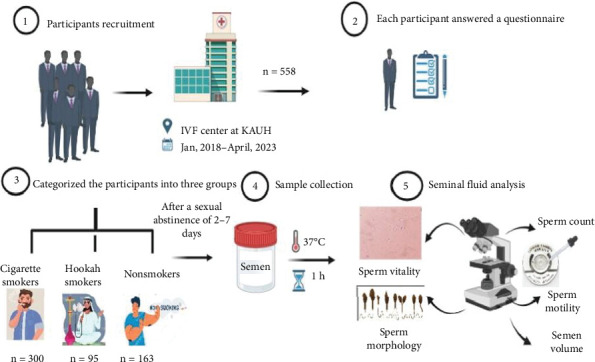
The conceptual study design of the present study.

The conceptual study design for the present work is illustrated in Figure 1. Each participant answered a questionnaire regarding his smoking habits. Recruited subjects were categorized into three groups: current cigarette smokers (*n* = 300), current hookah smokers (*n* = 95), and nonsmokers (*n* = 163). After a period of sexual abstinence lasting 2–7 days, semen samples were collected by masturbation in sterile containers made from polypropylene in a designated room adjacent to the andrology laboratory. Written and spoken instructions regarding semen collection were explained in detail to each participant.

### 2.2. Ethics Approval and Consent to Participate

Samples were collected from men who attended the andrology laboratory at the IVF Center at King Abdullah University Hospital, which is in accordance with the guiding principles of the Declaration of Helsinki. Prior to participation, each recruited subject signed an informed consent approved by the ethical committee of Jordan University of Science and Technology (IRB-IDs: 193-2018; 2022/152/12).

### 2.3. Semen Analysis

Collected samples were allowed to liquify within 1 h at 37°C. Semen samples with hyperviscosity were liquified by the addition of small portions (e.g., 10% or 20% of semen volume) of phosphate-buffered saline followed by repeated gentle pipetting (6–10 times) [18]. The dilution factor was considered in such abnormal samples when assessing sperm parameters, particularly count, motility, and vitality.

After 1 h of ejaculation, semen analysis was conducted following the WHO guidelines [18]. To ensure accurate and precise results, all sperm parameters were assessed in duplicate by expert andrologists who accomplished personal variation in the measurements of < 5%.

### 2.4. Measurement of Sperm Motility

A Makler counting chamber (Irvine Scientific, CA, United States) was used to assess motility and count sperm. A 10 *μ*L aliquot of semen was used to perform this test after gently vortexing the sample. The motility of sperm was categorized into progressive and nonprogressive based on the 2021 WHO guidelines [18]. Practically, highly active sperm that move linearly or circularly were considered progressive.

### 2.5. Measurement of Sperm Morphology

To ensure accurate morphology assessment, a ×10 eyepiece and a ×100 oil-immersion objective were utilized to obtain high-quality images under the microscope [18]. A minimum of 200 spermatozoa were assessed to reduce sampling error and enhance the reliability of the results.

### 2.6. Measurement of Sperm Vitality: Eosin Test

**Figure 2:**
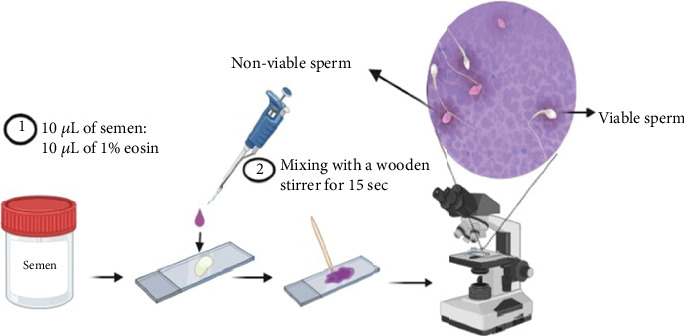
Vitality of sperm as assessed using eosin test. Viable spermatozoa retained their natural color (white), while nonviable spermatozoa exhibited a red hue due to eosin penetration through the damaged cell membrane.

In our analysis, we employed the Eosin test, a method specifically designed to distinguish between viable and nonviable spermatozoa based on their membrane integrity. To conduct the test, we combined 10 *μ*L of 1% aqueous eosin with 10 *μ*L of liquefied semen on a Boerner slide [18]. After thorough mixing with a wooden stirrer for approximately 15 s, the slides were covered with a coverslip and allowed to air-dry (Figure 2). Within this test, viable spermatozoa retained their natural color (white), while nonviable spermatozoa exhibited a red hue due to eosin penetration through the damaged cell membrane (Figure S1). Subsequently, we analyzed a total of 100 spermatozoa on each slide using a 400x objective lens [18].

### 2.7. Statistical Analysis

In this work, all data were analyzed using GraphPad Prism Version 9 (San Diego, United States). Semen parameters were represented as means ± standard errors of the means in all tested groups (cigarette smokers, hookah smokers, and nonsmokers). Ordinary one-way ANOVA followed by post hoc tests was used to compare the tested groups (cigarette smokers, hookah smokers, and nonsmokers). Compared means were considered statistically significant at a *p* value of less than 0.05, indicating that the observed differences are unlikely to have occurred by chance and are therefore likely to reflect a true effect in the population.

## 3. Results

**Figure 3:**
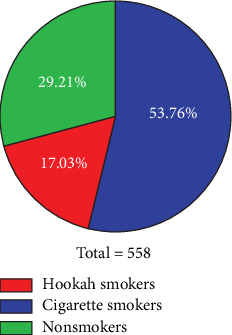
Prevalence of hookah and cigarette smoking among the participants (*n* = 558).

In this study, we examined and compared various characteristics related to semen quality, including semen volume, sperm motility (progressive and total), sperm count, sperm morphology, and sperm vitality. The comparison was made among three groups: hookah smokers, cigarette smokers, and nonsmokers.  Figure 3 displays the prevalence of hookah and cigarette smoking among the participants. Among cigarette smokers, the recruited participants had an average duration of smoking of approximately 14.18 ± 7.55 years, and the average daily cigarette consumption was approximately 23.92 ± 10.84 cigarettes. On the other hand, among hookah smokers, the average duration of smoking was approximately 11.5 ± 8.16 years, and the average frequency of hookah sessions per week was approximately 11.51 ± 8.16.

**Figure 4:**
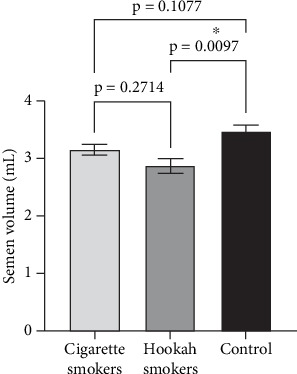
Effect of cigarette (*n* = 300) and hookah (*n* = 95) smoking on the semen volume compared to control (nonsmokers: *n* = 163) in all recruited subjects. Data are represented as means ± standard errors. Statistical analysis was performed using one-way ANOVA, followed by Tukey's post hoc test to compare group means. A significance level of *p* < 0.05 was considered statistically significant. ⁣^∗^*p* < 0.01.

Figure 4 demonstrates the effect of cigarette and hookah smoking on semen volume compared to control (nonsmokers). As shown in the figure, there is a significant difference in the ejaculated semen volume between hookah smokers (2.871 ± 0.150 mL) (*p* = 0.0122), but not cigarette smokers (3.152 ± 0.182 mL) (*p* = 0.1772), compared to control (3.457 ± 0.1996 mL). On the other hand, there was no significant difference (*p* = 0.2246) in the means of semen volumes between cigarette and hookah smokers. The percentage of reduction in the mean value of semen volume between hookah smokers and controls was approximately 16.4%.

**Figure 5:**
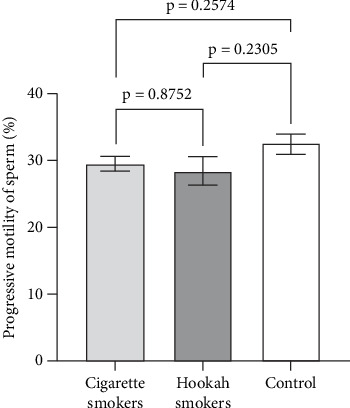
Effect of cigarette (*n* = 300) and hookah (*n* = 95) smoking on the progressive motility of sperm compared to control (nonsmokers: *n* = 163) in all recruited subjects (with normal motility and with asthenozoospermia). Data are represented as means ± standard error of the mean. Statistical analysis was performed using one-way ANOVA, followed by Tukey's post hoc test to compare group means. A significance level of *p* < 0.05 was considered statistically significant.

**Figure 6:**
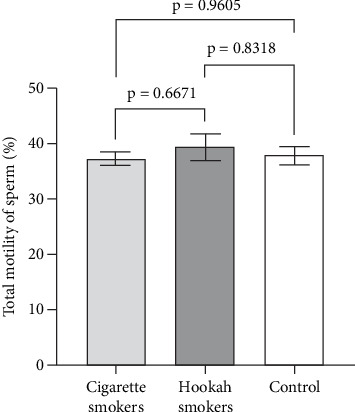
Effect of cigarette (*n* = 300) and hookah (*n* = 95) smoking on the total motility of sperm compared to control (nonsmokers: *n* = 163) in all recruited subjects. Data are represented as means ± standard error of the mean. Statistical analysis was performed using one-way ANOVA, followed by Tukey's post hoc test to compare group means. The significance level of *p* < 0.05 was considered statistically significant.

**Figure 7:**
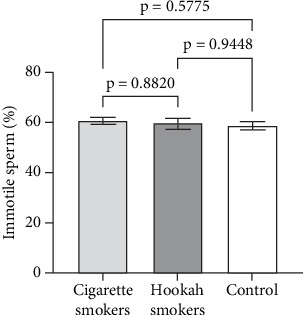
Effect of cigarette (*n* = 300) and hookah (*n* = 95) smoking on the percentage of immotile sperm compared to control (nonsmokers: *n* = 163) in all recruited subjects. Data are represented as means ± standard error of the mean. Statistical analysis was performed using one-way ANOVA, followed by Tukey's post hoc test to compare group means. A significance level of *p* < 0.05 was considered statistically significant.

Sperm motility is considered a sensitive parameter for assessing the impact of environmental exposures [19]. Therefore, in this study, we evaluated both the progressive and total motility of sperm, as well as the percentage of immotile sperm, to investigate the effects of hookah and cigarette smoking on sperm function. Figures 5, 6, and 7 illustrate the impact of cigarette smokers and hookah smokers on progressive motility, total motility, and immotile sperm, respectively. The figures demonstrate that there were no significant differences in the mean values of these parameters between the tested groups (cigarette: 29.51% ± 2.268%, 37.29% ± 2.468%, and 60.71% ± 2.446% vs. hookah: 28.4% ± 1.878%, 39.41% ± 2.043%, and 59.54% ± 2.027%, respectively) (*p* = 0.2714, *p* = 0.8752, and *p* = 6671, respectively), as well as when comparing each group to the control group (32.47% ± 2.487%, 37.84% ± 2.706%, and 58.68% ± 2.677%, respectively) (cigarette smokers vs. control: *p* = 0.2574, *p* = 0.9605, and *p* = 0.5775; hookah smokers vs. control: *p* = 0.2305, *p* = 0.8318, and *p* = 0.9448, respectively).

**Figure 8:**
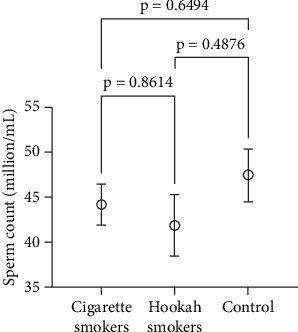
Effect of cigarette (*n* = 300) and hookah (*n* = 95) smoking on the sperm count compared to control (nonsmokers: *n* = 163) in all recruited subjects. Data are represented as means ± standard errors. Statistical analysis was performed using one-way ANOVA, followed by Tukey's post hoc test to compare group means. A significance level of *p* < 0.05 was considered statistically significant.

Sperm count is recognized as one of the crucial indicators of semen quality and can be affected by exposure to external toxic substances. Therefore, assessing sperm count provides valuable insights into the overall impact of smoking on sperm function.  Figure 8 presents the comparison of sperm count among cigarette smokers (44.20 ± 4.46 million/mL), hookah smokers (41.88 ± 3.700% million/mL), and the control group (47.48 ± 4.900% million/mL). Our findings indicate that there were no significant differences in sperm count between the tested groups (*p* = 0.8614) or when comparing each group to the control (*p* = 0.6494 and *p* = 0.4876, respectively).

**Figure 9:**
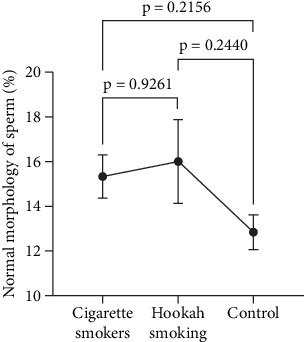
Effect of cigarette smoking (*n* = 300) and hookah smoking (*n* = 95) on the normal morphology of sperm compared to the control group (nonsmokers: *n* = 163) in the entire study population. The data are represented as means ± standard errors. Statistical analysis was performed using one-way ANOVA, followed by Tukey's post hoc test to compare group means. A significance level of *p* < 0.05 was considered statistically significant.

The next semen parameter we examined in relation to the effects of hookah and cigarette smoking is sperm morphology. In this study, we measured the percentage of normal sperm forms within each tested group. As demonstrated in Figure 9, similar to the previous findings, there were no significant differences observed in the percentage of normal sperm morphology between both types of smokers (cigarette vs. hookah: 15.34% ± 1.794% vs. 16.01% ± 1.485%) (*p* = 0.9261) and when comparing each type to the control group (12.85% ± 1.967%) (*p* = 0.2156 and *p* = 0.2440).

**Figure 10:**
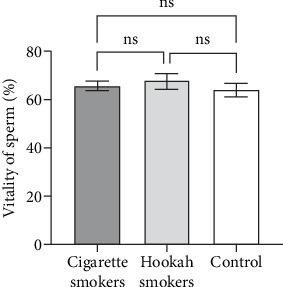
Effect of cigarette (*n* = 68) and hookah (*n* = 30) smoking on the vitality of sperm compared to control (nonsmokers: *n* = 54) in all recruited subjects. Data are represented as means ± standard errors. Statistical analysis was performed using one-way ANOVA, followed by Tukey's post hoc test to compare group means. A significance level of *p* < 0.05 was considered statistically significant.

Sperm vitality is defined as the assessment of the percentage of live and metabolically active sperm cells within a given semen sample. Also, it serves as a sensitive parameter to evaluate the influence of environmental exposures, particularly chemical toxicants. The final figure (Figure 10) presents the impact of cigarette and hookah smoking on sperm vitality in comparison to the control group. As depicted in the figure, there are no significant differences observed in the mean values between the tested groups (65.71% ± 3.946%, 67.67% ± 3.281%, and 64.07% ± 4.099%, respectively).

## 4. Discussion

In this study, we investigated the impact of hookah and cigarette smoking on essential human semen parameters, including semen volume, sperm count, sperm motility, sperm morphology, and sperm vitality. Additionally, this study represents the first attempt to compare these parameters between hookah smokers, cigarette smokers, and nonsmokers serving as the control group. The findings of this research revealed that hookah smokers exhibited a lower semen volume compared to the control group. However, no difference in semen volume was observed between cigarette smokers and the control group. Surprisingly, there was no difference in sperm count, percentage of sperm motility (both progressive and total), immotile sperm, normal forms of sperm, or sperm vitality between the two tested groups when compared to each other or to the control group.

The volume of ejaculated semen is mainly contributed by the male reproductive glands, particularly the seminal vesicles, providing approximately 65%–75% of ejaculate volume, and the prostate gland, contributing approximately 25%–30% of the volume [20, 21]. The bulbourethral glands contribute only a small volume (~ < 1%) [20]. Consequently, any exogenous or endogenous factors that affect these accessory glands may alter the volume of ejaculated semen. Therefore, evaluating the impact of hookah and cigarette smoking on semen volume appears to be a reasonable approach.

The cross-sectional study conducted by Albeitawi et al. in 2020 reported a trivial, yet statistically insignificant, effect of hookah smoking on semen volume [16]. One possible reason cited by the authors for this observation was the small sample size, which they recommended be increased for a more robust assessment of this correlation. In contrast, our study, which utilized a larger sample size from the studied group, demonstrated a significant impact of hookah smoking on semen volume.

Interestingly, semen volume was not found to be influenced by cigarette smoking [9], aligning with our findings in this study. Moreover, when comparing both types of smoking in terms of semen volume, the mean value of hookah smokers was approximately 10.4% lower than that of cigarette smokers. Pooled results from a meta-analysis indicated that semen volume was lower in heavy and moderate cigarette smokers compared to nonsmokers, while mild smokers exhibited higher volumes relative to these groups [22].

The observed differences in semen volume between the groups may be attributed to the distinctive exposure patterns and compositions of toxins in cigarette smoke versus hookah smoke. Hookah smoking typically involves prolonged exposure to higher quantities of smoke per session compared to cigarette smoking, potentially leading to more pronounced effects on semen volume. However, when comparing hookah smokers and cigarette smokers directly, the lack of a statistically significant difference might suggest that the detrimental effects of both types of smoking on semen volume converge over time, despite differences in exposure dynamics.

Furthermore, the apparent inconsistency could be due to variations in sample size, individual smoking behaviors (frequency and duration), or other confounding factors that were not fully controlled in our study. These factors might have contributed to the significant difference observed between hookah smokers and nonsmokers but not between cigarette smokers and hookah smokers.

Mechanistically, it has been suggested that tobacco consumption reduces the function of seminal vesicles and prostate glands. Evidence supporting this suggestion includes lower levels of total phosphate and N-acetyl amino sugar, which are markers of normal seminal vesicle function, as well as reduced levels of acid phosphatase and zinc, markers of normal prostate gland function, in tobacco smokers compared to nonsmokers.

Moreover, alpha-1,4-glucosidase, a marker of epididymis function, was also found to be significantly reduced in tobacco smokers compared to the control group. This reduction in the function of sex-accessory glands due to tobacco consumption provides clear evidence supporting the observed decrease in semen volume caused by the effect of hookah smoking. Indeed, a retrospective cross-sectional study involving 426 subjects demonstrated that smoking is associated with lower ejaculate volume and seminal vesicle volume [23].

Furthermore, it is widely acknowledged that cigarettes contain numerous chemical substances capable of inducing the generation of reactive oxygen species in body tissues [24–26]. This oxidative stress can lead to tissue injury and damage [27–29], ultimately impairing the normal function of these tissues. In particular, cigarette smoking has been shown to induce oxidative damage in the prostate gland, which can adversely affect its proper function [24, 30]. Notably, hookah tobacco also contains toxic constituents similar to those found in cigarettes, and as a result, cigarette and hookah smoking can have overlapping subjective effect measures. This suggests that the impact on seminal vesicles, prostate glands, and other sex-accessory glands may share common pathways between cigarette and hookah smoking due to the presence of similar harmful compounds in both types of tobacco products.

Nicotine is one of the most toxic substances found in tobacco smoke, and it has been detected in the semen of smokers [31, 32]. The toxicity of nicotine is attributed to its capacity to oxidize cell components, potentially leading to alterations in the intactness of plasma membranes and DNA integrity of sperm. Consequently, this oxidative damage can significantly impact sperm motility and overall function [33].

Furthermore, a study conducted by Alkhaled et al. revealed differences in sperm DNA methylation at various cytosine–phosphate–guanine dinucleotide sites within the protein tyrosine phosphatase receptor type N polypeptide 2 (PTPRN2) gene, phosphoglycerate mutase family member 5 (PGAM5) gene, and tyrosine–protein kinase receptor gene-related amplicons [34]. These differences in DNA methylation patterns may be associated with the effects of cigarettes or hookah smoking on the normal development of sperm. Such epigenetic changes could have implications for sperm function and may provide valuable insights into the impact of smoking on male reproductive health [34]. Additionally, a significant association was identified between alterations in sperm DNA methylation, particularly at certain cytosine–phosphate–guanine dinucleotide sites, and various sperm parameters, including count, motility, vitality, and morphology [34]. A recent study has revealed that, even though hookah smoking does not have a significant effect on semen parameters, it may have deleterious effects on oxidative status, nuclear protein levels, and DNA integrity of sperm [17].

Furthermore, research has revealed that smoking among normozoospermic individuals can lead to an increase in the production of nitric oxide, a free radical gas, and malondialdehyde, a marker of lipid peroxidation, in sperm. These changes in nitric oxide and malondialdehyde levels can significantly impact sperm function, particularly affecting sperm motility [35]. Consequently, smoking may have implications for male fertility by promoting nitric oxide production and elevating lipid peroxidation levels in sperm [35]. Understanding the effects of smoking on these biochemical processes in sperm is crucial for comprehending the mechanisms underlying smoking-related male reproductive health issues.

Alternatively, studies have demonstrated a significant decrease in testosterone levels with increasing smoking habit [36, 37]. Concurrently, Asare-Anane et al. reported a reduction in the volume of ejaculated semen in smokers compared to nonsmokers, alongside the reduced testosterone levels [37]. The precise mechanism by which smoking reduces testosterone levels is not yet fully understood, but it may involve direct chemical toxicity of smoking on Leydig cells [37], thereby adversely affecting testosterone synthesis. Consequently, the observed reduction in semen volume due to smoking, especially hookah smoking, may be attributed to a decline in testosterone biosynthesis. However, this mechanistic pathway warrants further investigation for a comprehensive understanding.

On the other hand, in the study conducted by Albeitawi et al., the effect of hookah smoking on progressive motility of sperm and sperm morphology was not found to be statistically significant [16]. Interestingly, these results align with those of our current study, demonstrating a consistent lack of significant impact of hookah smoking on these specific sperm parameters.

Alternatively, when examining the impact of cigarette smoking alone on semen parameters, a systematic review followed by a meta-analysis conducted by Sharma et al. revealed a correlation between exposure to cigarette smoking and a reduction in sperm morphology and count [22]. These results are not consistent with our findings. One possible reason for this inconsistency could be the sample size of our study and the specific nature of the selected population, which consists of northern Jordanians. In reality, different study populations may respond differently, to some extent, to exogenous toxicants due to variations in diet and lifestyle. Conducting multipopulational studies in this research context may yield more robust and comprehensive findings.

Additionally, a significant reduction in sperm count, progressive motility of sperm, normal forms of sperm, and sperm vitality was observed in infertile smokers compared to infertile [38]. Despite this, the general findings from that study are inconsistent with our current results. However, it is worth noting that we did observe a reduction, though statistically insignificant, in the mean percentage of progressive motility of sperm in our tested groups (hookah and cigarette smokers) compared to the control group. The differences in sample size and study population may be contributing factors behind this lack of statistical significance. Specifically, the tested group of hookah smokers is smaller than that of cigarette smokers and the control group, which presents a limitation in this study. Exploring larger sample sizes and diverse study populations in future research may help provide more comprehensive and conclusive results.

This study uniquely compares the effects of cigarette and hookah smoking, addressing a gap in existing research. In addition, the use of updated standardized guidelines [18] to measure semen quality characteristics ensures reliable and validated findings. However, the study has several limitations. Being cross-sectional, it captures data over a short period, limiting the ability to assess long-term effects. Designing a longitudinal study would provide better insights into the long-term impacts of cigarette and hookah smoking on semen quality. Additionally, observational studies like this are prone to potential confounding factors, such as lifestyle, diet, and environmental toxin exposure, which may influence the results. Furthermore, incorporating additional markers of sperm function, such as DNA fragmentation and oxidative stress, could offer a more comprehensive perspective. In conclusion, our study indicates that hookah smokers exhibit lower semen volume compared to the control group, while no significant differences were observed in sperm count, percentage of sperm motility (both total and progressive), and normal forms of sperm between hookah and cigarette smokers or between these smokers and nonsmokers. Nonetheless, the findings are limited by the sample size and specific study population. To establish more robust and comprehensive conclusions, a multipopulational long-term prospective study with large sample size is essential in this specific research context. Further investigations will help shed light on the potential impact of hookah smoking on male reproductive health and fertility.

## Data Availability

The data that support the findings of this study are available from the corresponding author upon reasonable request.

## Supporting Information

Supporting Information Additional supporting information can be found online in the Supporting Information section. Figure S1: Vitality of sperm as assessed via eosin test. Viable spermatozoa retained their natural color (white), while nonviable spermatozoa exhibited a red hue due to eosin penetration through the damaged cell membrane.
