# Supplementary material for: Semen Quality Measures in Hookah and Cigarette Smokers Compared to Nonsmokers
Source: ScientificWorldJournal. 2025 Feb 17;2025:3380445. doi: 10.1155/tswj/3380445 (PMC11850069; doi:10.1155/tswj/3380445)
Supplement: Supporting Information — Additional supporting information can be found online in the Supporting Information section. Figure S1: Vitality of sperm as assessed via eosin test. Viable spermatozoa retained their natural color (white), while nonviable spermatozoa exhibited a red hue due to eosin penetration through the damaged cell membrane. [file 3380445.f1.pdf]

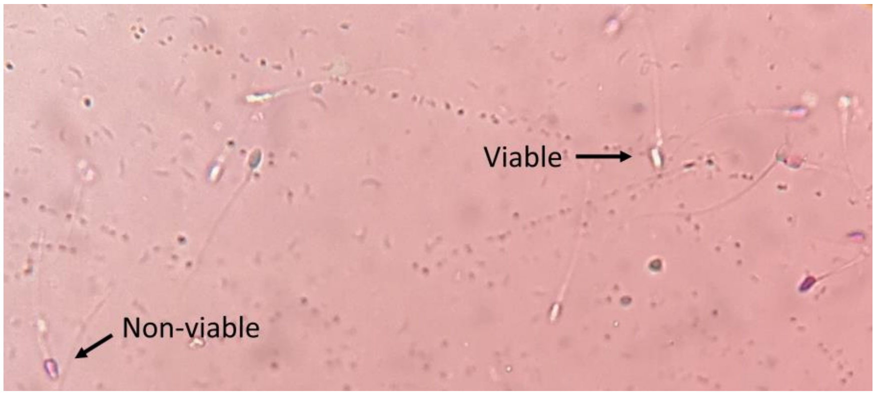

Supporting Figure 1: Vitality of sperm as assessed via Eosin test. Viable spermatozoa retained their natural color (white), while non-viable spermatozoa exhibited a red hue due to eosin penetration through the damaged cell membrane.
